# Supplementary material for: A large, comprehensive labeled MRI dataset of the human lateral ventricle choroid plexus
Source: bioRxiv. 2026 Oct 11:2026.10.08.757728. Preprint. [Version 1] doi: 10.64898/2026.10.08.757728 (PMC13692223; doi:10.64898/2026.10.08.757728)

bioRxiv preprint doi: https://doi.org/10.64898/2026.10.08.757728; this version posted October 11, 2026. The copyright holder for this preprint (which was not certified by peer review) is the author/funder, who has granted bioRxiv a license to display the preprint in perpetuity. It is made available under a CC-BY 4.0 International license.

Figure S1. Step-by-step tutorial for manual segmentation of human lateral ventricle ChP using 3D Slicer. This protocol is followed throughout DataPlex construction.

Step 1: Load the image into the graphical interface.

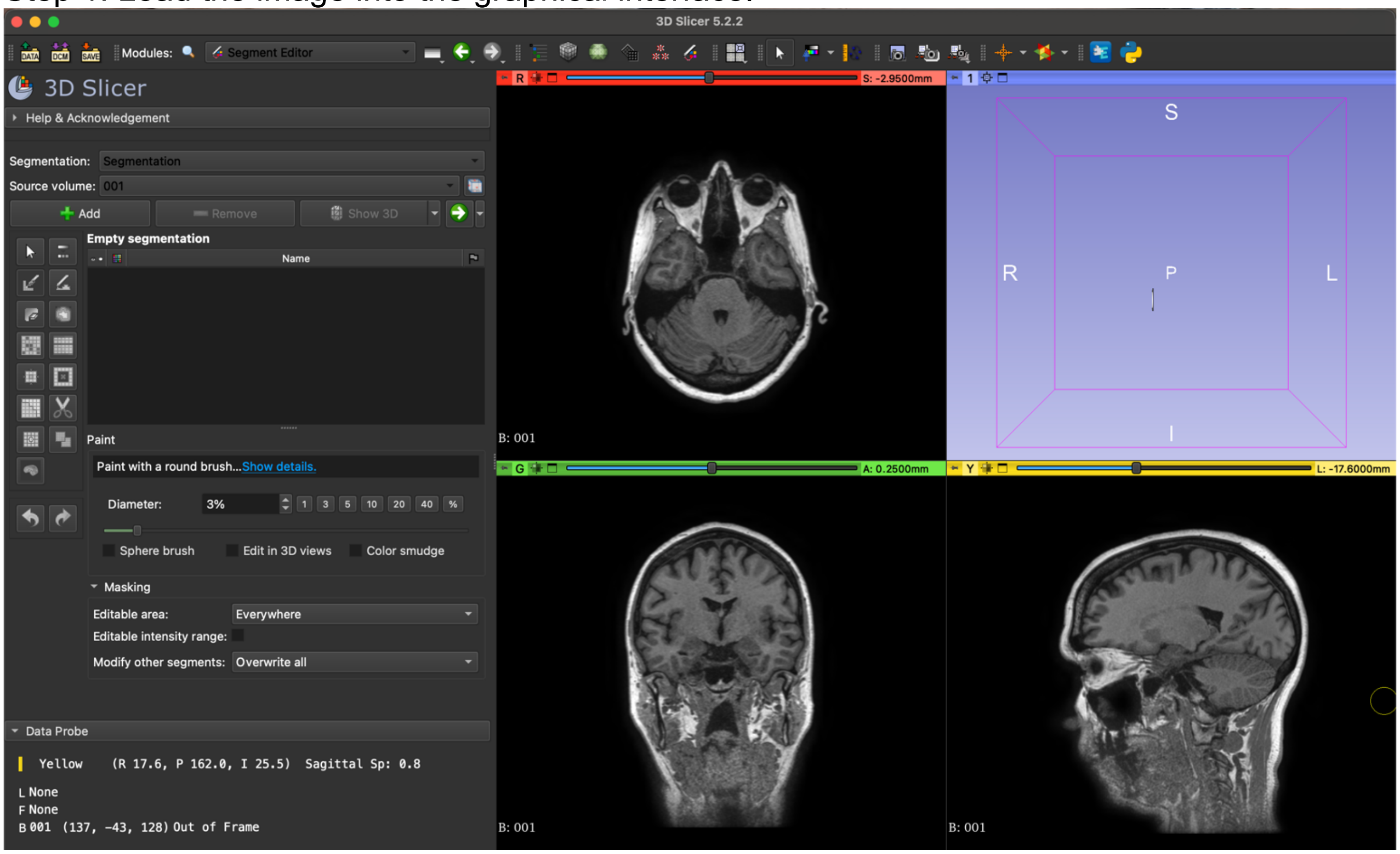

Step 2: Create two segmentation masks.

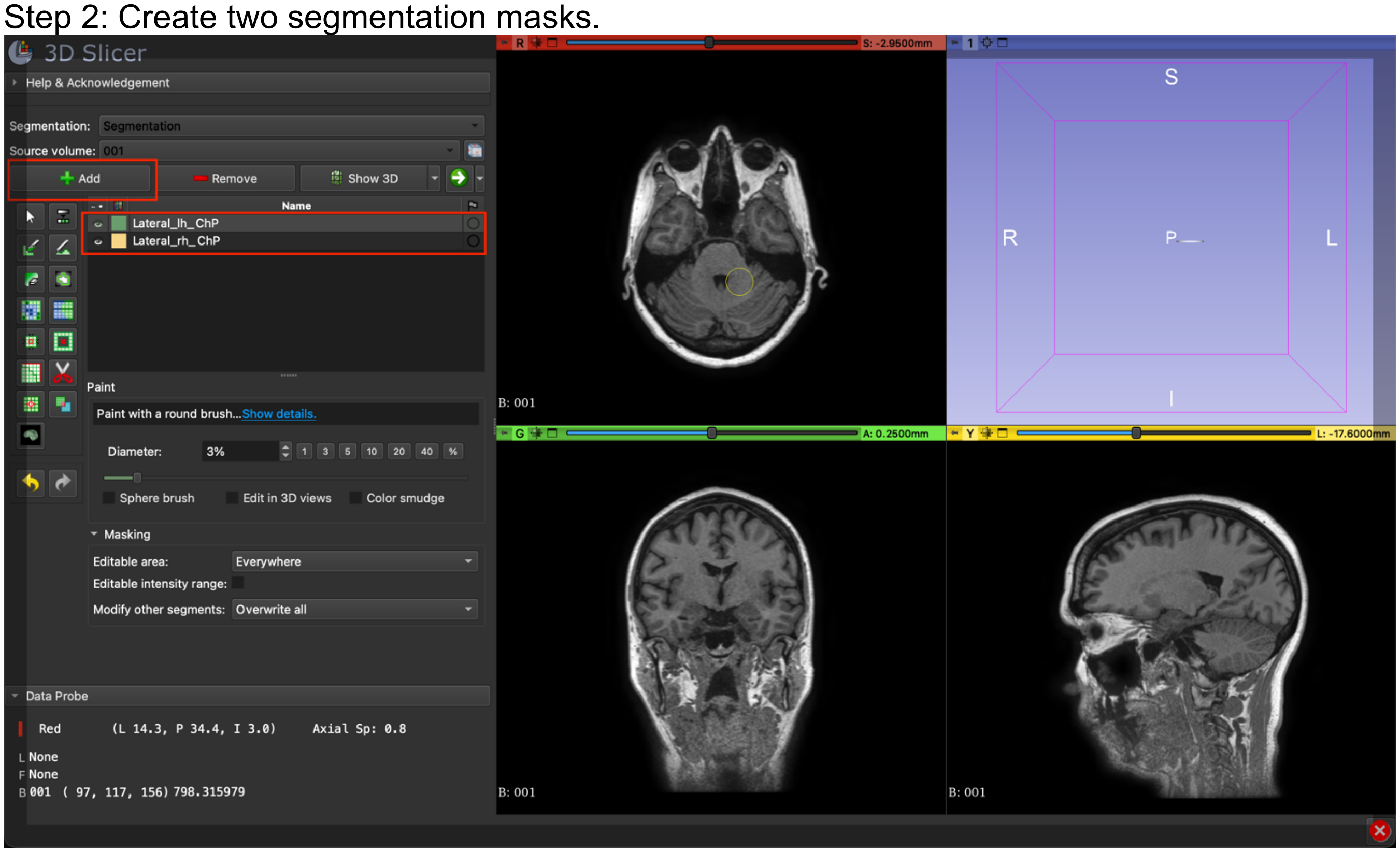

bioRxiv preprint doi: https://doi.org/10.64898/2026.10.08.757728; this version posted October 11, 2026. The copyright holder for this preprint (which was not certified by peer review) is the author/funder, who has granted bioRxiv a license to display the preprint in perpetuity. It is made available under a CC-BY 4.0 International license.

Step 3: Use windowing and anatomical landmarks to maximize tissue contrast for clear ChP identification: 1) ensure that the aqueous ventricles appear dark, and 2) ensure the striatal area show clear grey/white matter contrast between the caudate and the internal capsule.

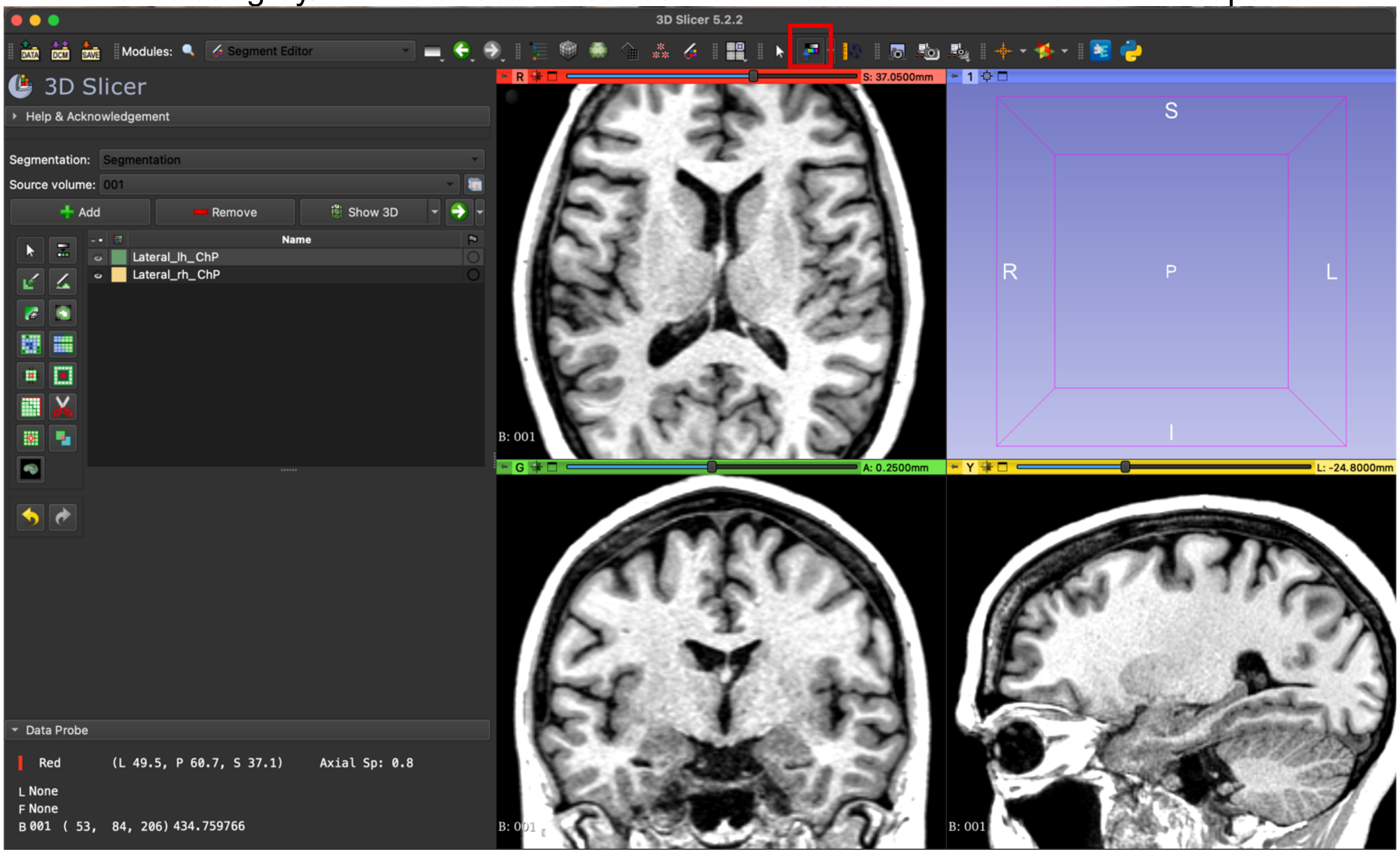

Step 4: Remove brush fill in segmentation settings.

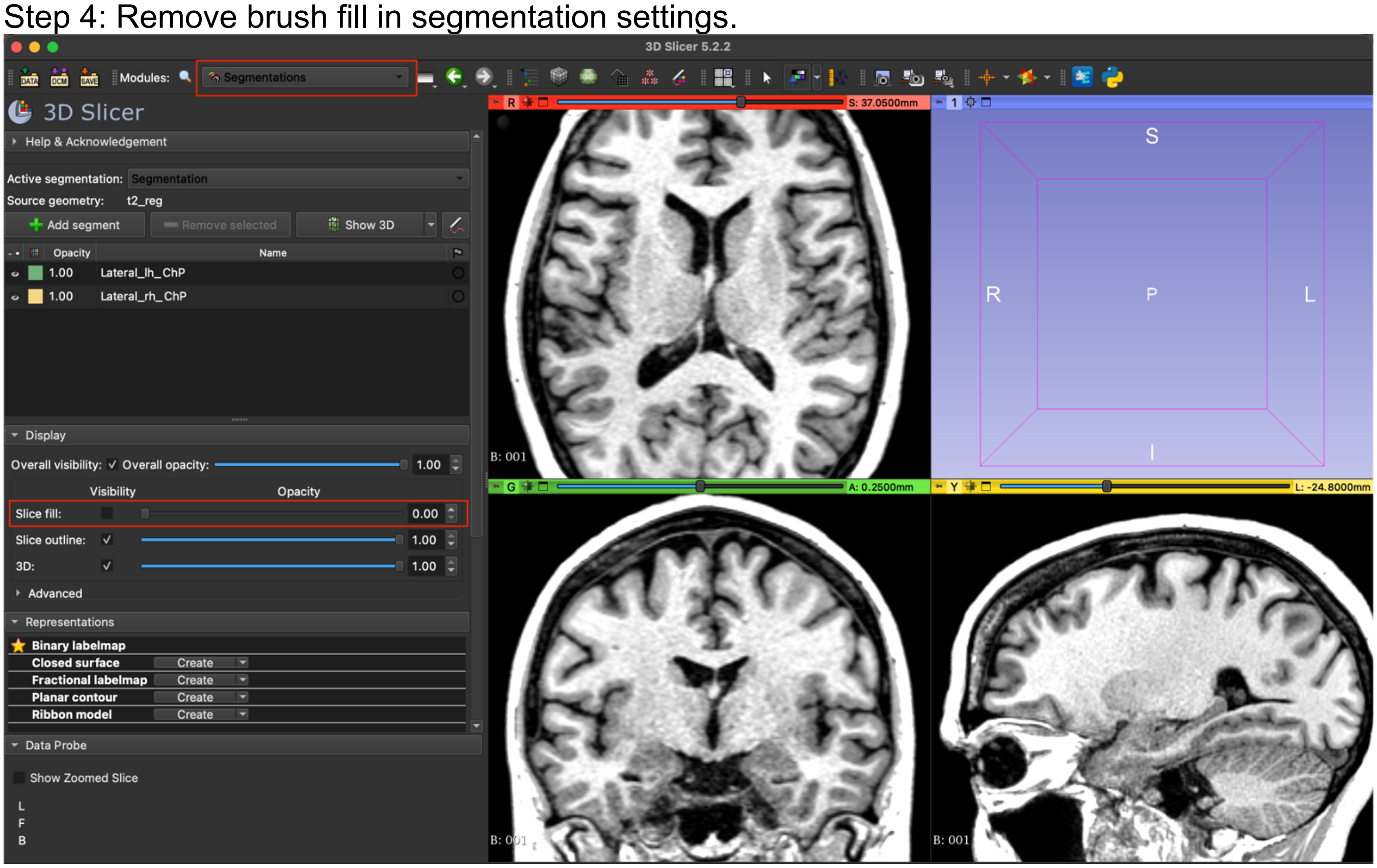

bioRxiv preprint doi: https://doi.org/10.64898/2026.10.08.757728; this version posted October 11, 2026. The copyright holder for this preprint (which was not certified by peer review) is the author/funder, who has granted bioRxiv a license to display the preprint in perpetuity. It is made available under a CC-BY 4.0 International license.

Step 5: Select the finest brush size.

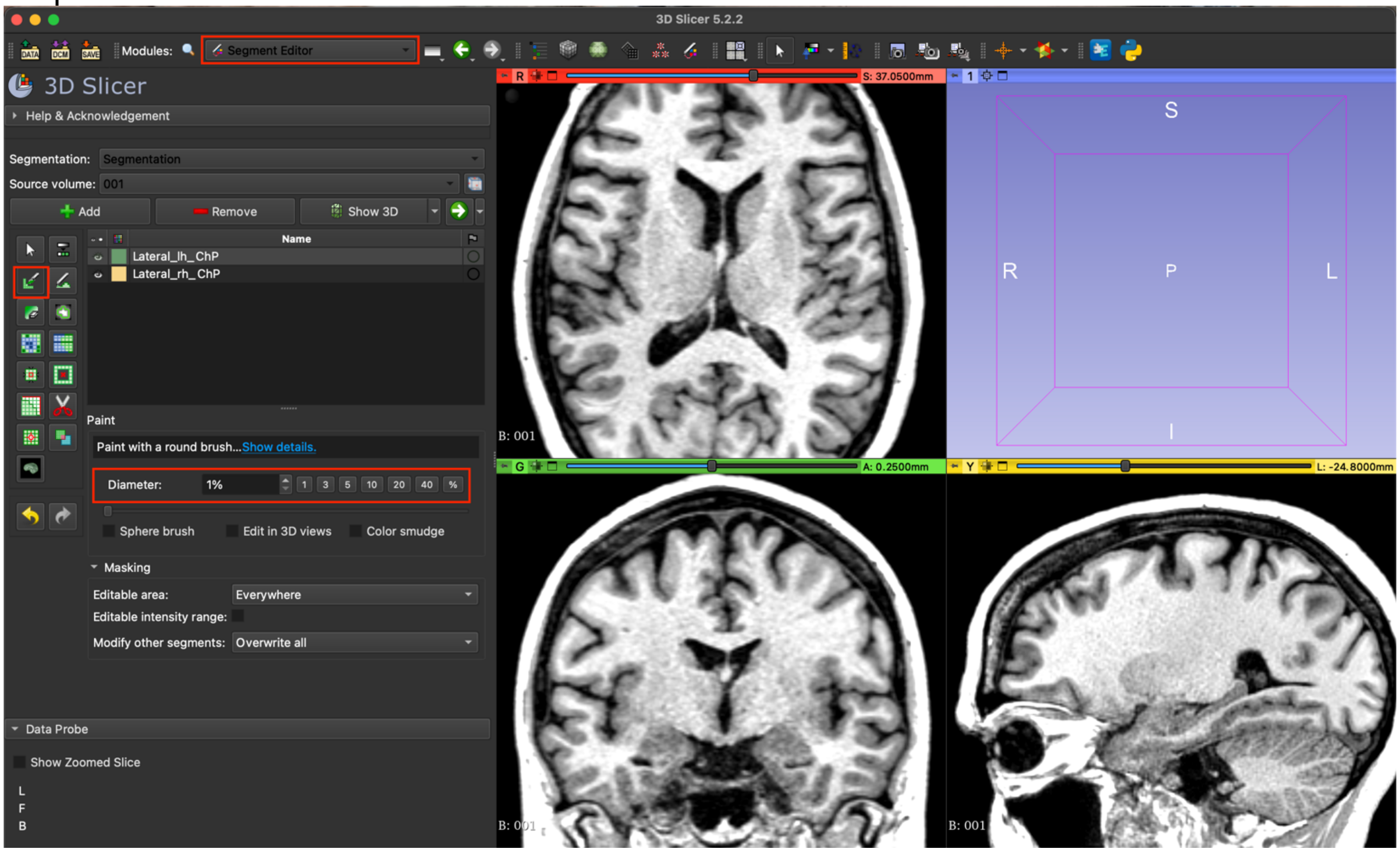

Step 6: Anchor the first few labeled voxels of the left hemisphere (right side in the radiological orientation) at the apical edge of ChP (red arrows). Slicer allows location lock (yellow rectangle) for viewing the same voxel in all 3 views simultaneously. Repeat 6-8 on both sides.

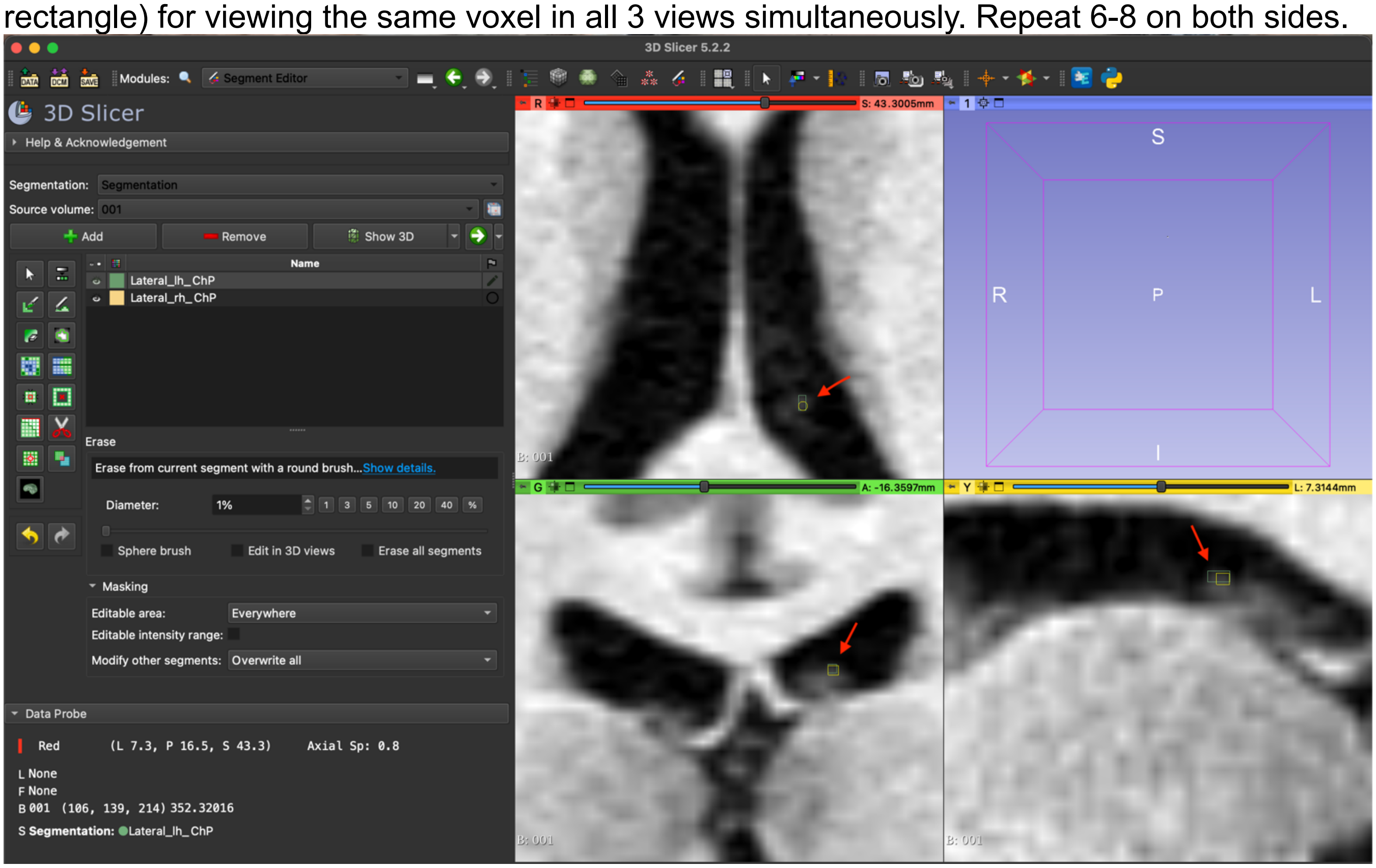

bioRxiv preprint doi: https://doi.org/10.64898/2026.10.08.757728; this version posted October 11, 2026. The copyright holder for this preprint (which was not certified by peer review) is the author/funder, who has granted bioRxiv a license to display the preprint in perpetuity. It is made available under a CC-BY 4.0 International license.

Step 7: Find the anchored voxels on the sagittal view using the location lock and label the rest of ChP on the slice

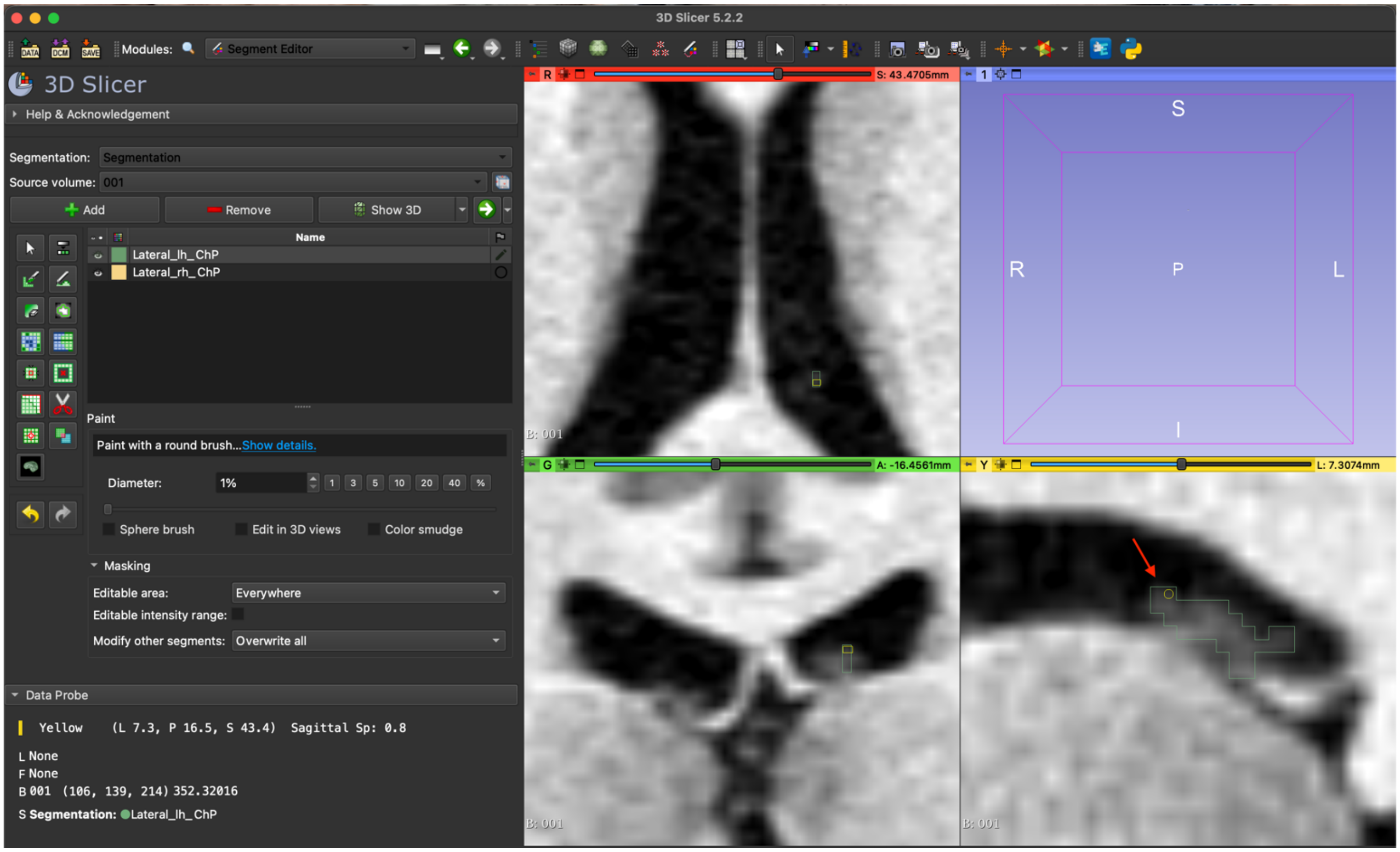

Step 8: Continue segmenting on the sagittal view. When all sagittal slices are completed, scroll through the axial and coronal views to ensure completeness and accuracy.

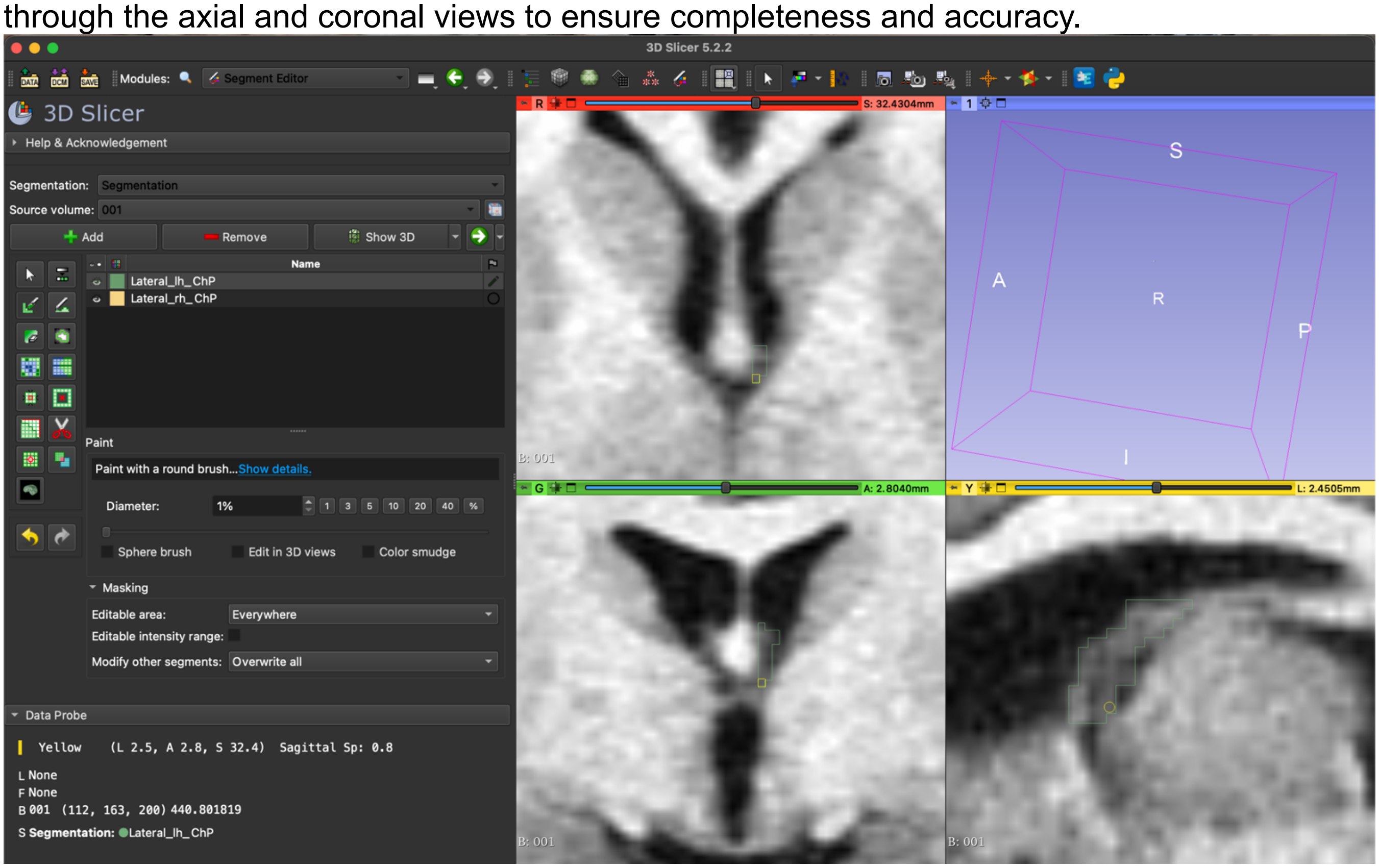

bioRxiv preprint doi: https://doi.org/10.64898/2026.10.08.757728; this version posted October 11, 2026. The copyright holder for this preprint (which was not certified by peer review) is the author/funder, who has granted bioRxiv a license to display the preprint in perpetuity. It is made available under a CC-BY 4.0 International license.

Figure S2: T2-weighted images highlight vasculature and aids manual segmentation of lateral ventricle ChP. This figure shows a direct comparison of T1-weighted and T2-weighted images surrounding the same voxel. The T1 image slices surrounding the same voxel (yellow square, pointing to the striatal-thalamic vein) are displayed in the top row, and the T2 images are on the bottom. Left to right: axial, coronal, and sagittal views.

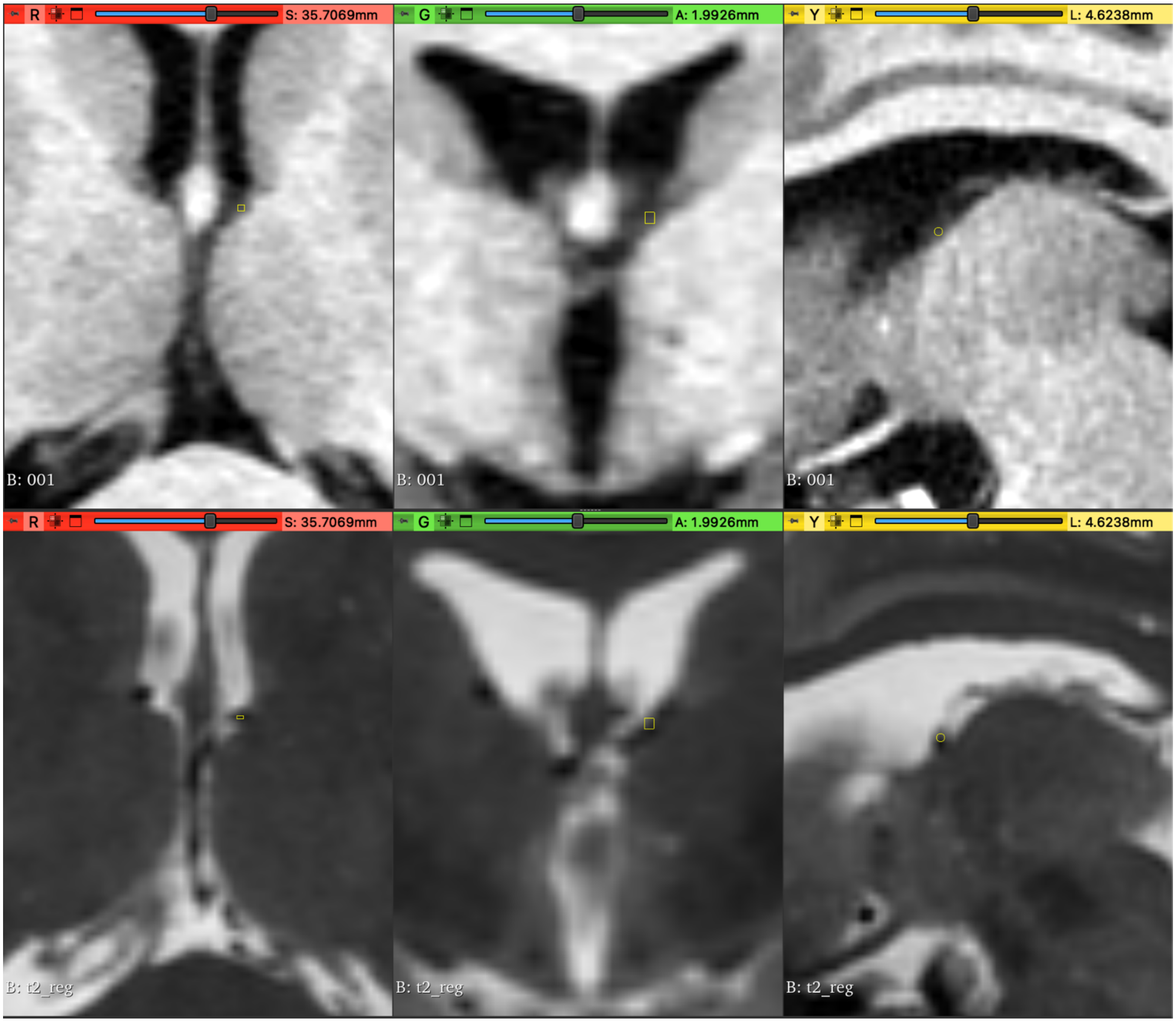

Supplement: Supplementary file 1 [file NIHPP2026.10.08.757728v1-supplement-1.pdf]
